# Nestin Is Essential for Zebrafish Brain and Eye Development through Control of Progenitor Cell Apoptosis

**DOI:** 10.1371/journal.pone.0009318

**Published:** 2010-02-19

**Authors:** Hua-Ling Chen, Chiou-Hwa Yuh, Kenneth K. Wu

**Affiliations:** 1 Institute of Cellular and System Medicine, National Health Research Institutes, Zhunan, Taiwan; 2 Division of Molecular and Genomic Medicine, National Health Research Institutes, Zhunan, Taiwan; Fred Hutchinson Cancer Research Center, United States of America

## Abstract

**Background:**

Nestin is expressed in neural progenitor cells (NPC) of developing brain. Despite its wide use as an NPC marker, the function of nestin in embryo development is unclear.

**Methodology/Principal Findings:**

As *nestin* is conserved in zebrafish and its predicted sequence is clustered with the mammalian *nestin* orthologue, we used zebrafish as a model to investigate its role in embryogenesis. Injection of *nestin* morpholino (MO) into fertilized eggs induced time- and dose-dependent brain and eye developmental defects. *Nestin* morphants exhibited characteristic morphological changes including small head, small eyes and hydrocephalus. Histological examinations show reduced hind- and mid-brain size, dilated ventricle, poorly organized retina and underdeveloped lens. Injection of control *nestin* MO did not induce brain or eye changes. *Nestin* MO injection reduced expression of *ascl1b (achaete-scute complex-like 1b)*, a marker of NPCs, without affecting its distribution. *Nestin* MO did not influence Elavl3/4 (Embryonic lethal, abnormal vision, Drosophila-like 3/4) (a neuronal marker), or *otx2* (a midbrain neuronal marker), but severely perturbed cranial motor nerve development and axon distribution. To determine whether the developmental defects are due to excessive NPC apoptosis and/or reduced NPC proliferation, we analyzed apoptosis by TUNEL assay and acridine orange staining and proliferation by BrdU incorporation, *pcna* and *mcm5* expressions. Excessive apoptosis was noted in hindbrain and midbrain cells. Apoptotic signals were colocalized with *ascl1b*. Proliferation markers were not significantly altered by *nestin* MO.

**Conclusion/Significance:**

These results suggest that nestin is essential for zebrafish brain and eye development probably through control of progenitor cell apoptosis.

## Introduction

Nestin is an intermediate filament protein which exhibits structural similarities to vimentin, desmin and neuro-filaments and is classified as a type IV neuro-filament [1]. Nestin is assembled into intermediate filaments by forming heterodimers with vimentin and desmin [2], [3] and together with microtubules and microfilaments it forms the cytoskeleton. Nestin was originally cloned from the central nervous system of rat embryos [1]. Its expression was found primarily in neuroepithelial stem cells [1] and proliferating neural progenitor cells (NPC) [4]. The expression of *nestin* can also be found in the parenchyma [5]. In developing murine embryos, *nestin* expression coincides with the onset of neurulation and is dramatically down-regulated in mature neural cells [1]. *Nestin* is conserved across diverse species including mammals, chicken and zebrafish. Phylogenetic analysis reveals that the predicted amino acid sequence of zebrafish *nestin* is clustered with the mammalian *nesti*n orthologue [6]. Furthermore, similar to mammals, zebrafish nestin is expressed widely in developing nervous system and its expression is restricted to neural proliferation zone in adult fish [6], [7].

Despite extensive investigations to ascertain *nestin* as a marker of neural stem and progenitor cells, the functions of nestin are not clearly understood. Recent reports suggest that nestin may be involved in positioning and functioning of subcellular organelles and may serve as a scaffold for kinases that regulate diverse cellular functions such as proliferation, survival and differentiation [8], [9], [10], [11]. However, it is unclear whether nestin is important in neural development. In this study, we evaluated the function of nestin in zebrafish embryogenesis by injecting *nestin* morpholino (MO) into fertilized eggs. *Nestin* MO treatment resulted in brain and eye developmental defects. These developmental defects were accompanied by reduction in NPCs and increase in neural and retinal apoptosis.

## Materials and Methods

### Zebrafish Husbandry, Experimentation and Care/Welfare

AB strain fish *Danio rerio* and transgenic *Tg(gfap:GFP)* fish were purchased from Zebrafish International Resource Center (ZIRC), Oregon. *Tg(isl1:GFP)* line was kindly provided by Dr. Hitoshi Okamoto [12]. Heterozygous *gfap:GFP* was generated by crossing *Tg(gfap:GFP)* with wild type AB strain. All the fish were maintained at 28°C under continuous flow in our zebrafish facility with automatic control for a 14-hour light and 10-hour dark cycle.

To generate embryos for injection, male and female fish were placed the night before injection in a one liter fish tank with the inner mesh and divider. Zebrafish embryos were obtained from natural spawning by removing the divider and light stimulation. The embryos were kept at 28°C before and after microinjection. All the experiments involving zebrafish had been conducted according to the guidelines of Institutional Animal Care and Use Committee (IACUC) of National Health Research Institutes (NHRI). The zebrafish experimental protocols were approved by IACUC (approval number: NHRI-IACUC-095050-A, 096037-A, 098017-A and 098087 under Dr. Chiou-Hwa Yuh).

### Morpholino (MO) Injections

Zebrafish standard control MO (5′-CCTCTTACCTCAGTTACAATTTATA-3′), *nestin* MO1 (5′-CGAGAGATATGAAGTGAAATCTCAC-3′), 5-bp mismatch (5-mis) *nestin* MO1 control (5′-CGAcAGATATcAAcTGAAATgTgAC-3′, lower case letters indicate mismatch bases), and *nestin* MO2 (5′-TGTCGGACACCCAGAAGCTCCATCT-3′) were purchased from Gene Tools, LLC (Philomath, OR), dissolved to 4.12 µg/µl in sterilized ddH_2_O and stored at −20°C. For microinjection, MOs were prepared in 1x PBS with 0.05% phenol red. They were injected into embryos of one-cell stage with PV820 Pneumatic PicoPump (World Precision Instruments, Inc., Sarasota, FL). After injection, embryos were incubated in egg water (60 µg sea salt/ml distilled water) supplemented with 0.003% 1-phenyl-2-thiourea (PTU) at 28°C to prevent pigment formation. Embryo development was evaluated at approximately 24 hpf, 48 hpf, and 72 hpf.

### DNA Construction, Microinjection and Western Blot Analysis

To evaluate the knockdown efficiency of *nestin* MO1, we amplified by PCR a 330 bp fragment of zebrafish nestin cDNA (−58 to +272, +1 indicates translation initiation site) encompassing MO1 targeting sequence (−28 to −4) using primers: *nestin*-XhoF (5′-AATA**CTCGAG**TCAAAGTGCCCCATTTCTTC-3′, bold letters are *Xho*I site) and *nestin*-KpnR (5′-AATA**GGTACC** TCTACCTCCAGCTCCACACA-3′, bold letters are *Kpn*I site), and cloned it into pEGFP-N1 plasmid. 200 pg of linealized *nestin*-GFP plasmid was microinjected into one-cell stage embryos, together with 10 ng of *nestin* MO1 or 5-mis *nestin* MO1. Embryos were harvested at 30 hpf and lysed with radio-immunoprecipitation assay buffer plus a complete protease inhibitor cocktail (Roche, Mannheim, Germany), and GFP was analyzed by Western blotting. In brief, 100 µg proteins were loaded onto each lane. Rabbit anti-GFP antibody (1∶500, Santa Cruz, Santa Cruz, CA) and mouse anti-β actin antibody (Sigma-Aldrich, St. Louis, MO) were used for GFP protein detection and internal control. Goat anti-rabbit and goat anti-mouse IgG conjugated with horseradish peroxidase (Santa Cruz) were used in protein detection. Protein bands were visualized by enhanced chemiluminescence (Pierce Chemical, Rockford, IL).

### Bright Light and Fluorescent Microscopy

Embryos were anesthetized with 0.168 mg/ml tricaine (Sigma-Aldrich), mounted in 2% methylcellulose and examined on a Leica fluorescent microscope (DMIRB) equipped with a CoolSNAP TM cooled CCD camera (Roper Scientific, Trenton, NJ).

### Histologic Sections and H&E Staining

Embryos were fixed with 4% paraformaldehyde overnight at 4°C and mounted in 1% agarose. The agarose block was gradually dehydrated with 70%, 90%, 100% ethanol and 100% xylene and embedded in paraffin [13]. The brain was cut into 7 µm sections which were stained with hematoxylin and eosin (H&E stain).

### Whole Mount *In Situ* Hybridization (WMISH)

Embryos were fixed by 4% paraformaldehyde overnight at 4°C and dehydrated in methanol at −20°C. WMISH procedure was performed according to the protocol described in detail by Thisse B and Thisse C on ZFIN (http://zfin.org) with some modifications. In brief, embryos were rehydrated gradually with PBST (1x PBS, 0.1% Tween 20). For 25-hpf embryos, they were digested with 10 µg/ml proteinase K (Sigma-Aldrich) in PBST for 12 minutes at room temperature. For 49-hr embryos, they were treated with proteinase K for 30 minutes at 30°C. After postfixed with 4% paraformaldehyde, the embryos were incubated in HYB^+^ (50 µg/ml heparin, 500 µg/ml wheat germ tRNA and HYB^−^ containing 50% formamide, 5x SSC and 0.1% Tween 20) which contained 100 ng DIG-labeled antisense RNA probe at 60°C overnight. Embryos were then washed with 75%, 50% and 25% HYB^−^/2x SSC for 15 minutes, 2x SSC for 15 minutes and two times with 0.2x SSC for 30 minutes at 65°C and transferred gradually to 2x blocking buffer (2% Blocking Reagent (Roche) in 100 mM maleic acid, 150 mM NaCl, and 0.1% Tween 20) at least for 1 hour. Embryos were incubated in 1∶5000 anti-DIG-AP (Roche) in 2x blocking buffer overnight at 4°C. After 6 washes with PBST for 15 minutes and 3 washes with alkaline Tris buffer for 5 minutes, bound antibody was detected by SIGMA *FAST*™ BCIP/NBT. After staining, labeled embryos were mounted in 90% glycerol and examined on an Olympus stereomicroscope (SZX-ILLD100, Tokyo, Japan). The images were captured using an Olympus DP70 digital microscope camera and processed by Helicon Focus software. The sequences of primers used in synthesizing probes are as follows: *ascl1b*-forward (F): 5′-ACGACGCAACTGACACAAGA-3′ and reverse (R): 5′-GGAACACACTGGCTCATTCA-3′; *isl1*-F: 5′-CACAGATAAAAACACGGGCA-3′ and R: 5′-GGAACGAAACAAAACCTCCA-3′; *mcm5*-F: 5′-GCAAGAATGTCGGGATTTGA-3′ and R: 5′-GAGGAACGGCATCAGGAG-3′; *nestin*-F: 5′-GTACCAGATGCTAGAGCTGAAC-3′ and R: 5′-GCATCTGCCTCTTGATCCTC-3′; *otx2*-F: 5′-AAAAGAGACCGAAGCAGCC-3′ and R: 5′-ATACGCATTCACGACCCTTC-3′; and *pcna*-F: 5′-GGCTTGCTGGGATGTGAG-3′ and R: 5′-TGGCTTTAGAGGGTCACAATCGG-3′.

### Whole-Mount Immunohistochemistry

For Elavl3/4 staining, embryos were fixed in 4% paraformaldehyde overnight at 4°C and dehydrated in methanol at −20°C. For acetylated tubulin staining, embryos were fixed in Dent's fixative (80% methanol, 20% DMSO) overnight at 4°C. The embryos were permeabilized with proteinase K followed by post-fixation with 4% paraformaldehyde, and washed with PBSTX (PBS +0.5% Triton X-100). After treating with 4% normal goat serum (NGS) in PBSTX for 2 hours at room temperature, embryos were incubated with mouse anti-Elavl3/4 antibody (1∶500, Molecular Probes, Eugene, OR) or mouse anti-acetylated tubulin antibody (1∶1000, Sigma-Aldrich) in 4% NGS/PBSTX overnight at 4°C. The embryos were washed five times with PBSTX for 10 minutes each and incubated with goat anti-mouse FITC (1∶200, Jackson ImmunoReseasrch, West Grove, PA) in 2% NGS/PBSTX for 2 hours at room temperature. After washing 5 times for 10 minutes each, embryos were mounted in 1% agarose and examined by confocal microscopy (Olympus Fluoview FV300).

For BrdU incorporation, 24 hpf embryos were treated with 10 mM BrdU in 15% DMSO/egg water for 20 minutes at 4°C and washed three times with egg water for 5 minutes each, followed by 4% paraformaldehyde fixation overnight at 4°C and dehydration in methanol at −20°C. After gradual rehydration, embryos were permeabilized with proteinase K followed by post-fixation with 4% paraformaldehyde, washed with PBSTX, blocked with 4% NGS in PBSTX for at least 2 hours at room temperature, and incubated with rat anti-BrdU-FITC antibody (1∶200, Abcam, Cambridge, MA) in 4% NGS/PBSTX overnight at 4°C. After washing five times with PBSTX for 10 minutes each, they were mounted in 1% agarose and examined by confocal microscopy.

### Detection of Cell Death

To detect apoptotic cells in live embryos, embryos were dechorionated and soaked in egg water containing 2 µg/ml acridine orange at 28°C for 30 minutes. After washing with egg water eight times 5 minutes each, embryos were anesthetized with tricaine, mounted in 2% methylcellulose and examined by stereomicroscopy, or confocal microscopy.

Apoptotic cells were also examined by TUNEL assay. Embryos were fixed with 4% paraformaldehyde overnight at 4°C and dehydrated with methanol at −20°C. After gradual rehydration, the embryos were permeabilized with 25 µg/ml proteinase K for 30 minutes at 30°C followed by 4% paraformaldehyde, and incubated with 90 µl labeling solution plus 10 µl enzyme solution (In Situ Cell Death Detection Kit, Fluorescein, Roche) at 37°C for 2 hours. They were washed three times with PBST for 5 minutes each and the images were examined by confocal microscopy.

To determine whether apoptosis was localized to neural precursor cells, embryos were first *in situ* hybridized with fluorescence *ascl1b* RNA probe followed by TUNEL assay. The protocol of fluorescence *in situ* hybridization is as described above with some modifications [14]. The RNA probe was labeled by dinitrophenyl (DNP). After conjugated with anti-DNP-POD antibody (PerkinElmer, Shelton, CT), the signal was detected by TSA-fluorescein system (PerkinElmer). This was followed by TUNEL assay, using TMR red staining (Roche). The embryos were manually de-yolked and the images were captured on an Olympus confocal microscope.

## Results

### Brain and Eye Developmental Defects Induced by Nestin MO

**Figure 1:**
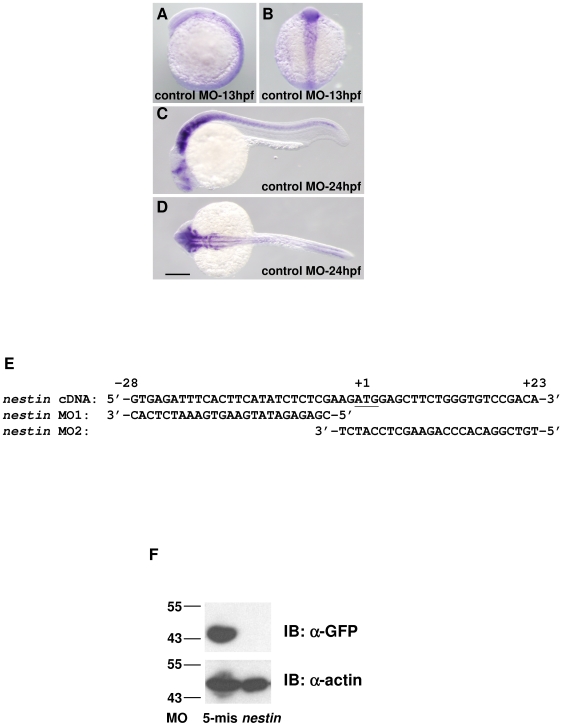
*Nestin* expression in zebrafish embryos. ***A–D***. Analysis of *nestin* transcripts by whole-mount *in situ* hybridization in embryos injected with control MO. ***A*** and ***C***, lateral view from left; ***B***, dorsal view, head at top and ***D***, dorsal view, head at left. Scale bar: 200 µm. ***E***. Nucleotide sequence around the translation start site of zebrafish nestin cDNA and corresponding sequence of *nestin* MO1 and MO2. ***F***. Western blot analysis of nestin:GFP fusion proteins in 30 hpf embryos treated with *nestin* MO (10 ng) or its 5-mis MO control (10 ng). The proteins were immunoblotted (IB) with a GFP (α-GFP) or actin antibody (α-actin).

*Nestin* expression was detected in embryos at 13 hpf (Fig. 1A and 1B) and was confined to nervous system at 24 hpf (Fig. 1C and 1D). To determine the role of nestin in embryo development, we tested several *nestin* MO sequences and identified *nestin* MO1 and MO2 to be active. Their sequences relative to nestin cDNA are shown in Fig. 1E. To test the knockdown efficiency, *nestin* MO1 or its 5-mis MO control was co-injected with *nestin-GFP* plasmids into fertilized eggs. Nestin-GFP fusion proteins analyzed in 30 hpf embryos became undetectable after *nestin* MO treatment (Fig. 1F). The 5-mis control did not suppress nestin-GFP proteins (Fig. 1F).

**Figure 2:**
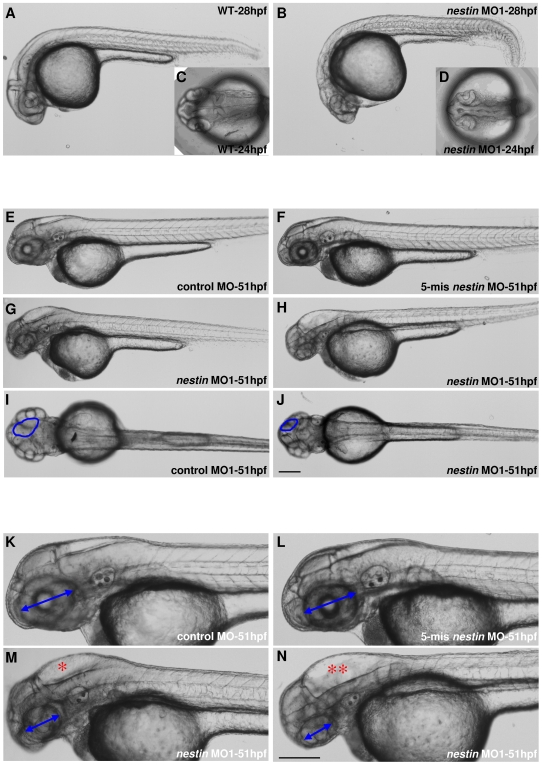
Embryo morphological changes induced by *nestin* MO. ***A–D***. One-cell stage embryos were injected with 10 ng *nestin* MO and morphology was examined at 24 hpf and 28 hpf. Compared to normal morphology of wild-type (WT) (***A***: lateral view and ***C***: dorsal view), *nestin* morphant exhibited head malformation (***B***: lateral view) with unclear boundaries between brain subdivisions, especially the hindbrain and midbrain boundary (***D***: dorsal view). ***E-J***. Embryos were examined at 51 hpf after injection of 10 ng *nestin* MO1 or an equal amount of control MO and 5-mis *nestin* MO. The head abnormality had become more pronounced in *nestin* morphants with overt hydrocephalus (***G*** and ***H***). ***G*** illustrates mild and ***H***, severe hydrocephalus. The morphology of control MO and 5-mis *nestin* MO remained normal (***E*** and ***F***). Dorsal view of *nestin* morphants revealed reduced size of tectum (***J***) compared with control morphants (***I***). ***K–N***. Magnified views of ***E–H*** to illustrate the gross head and eye defects and hydrocephalus in *nestin* morphants. Blue circles in ***I*** and ***J*** refer to tectum. Blue double-head arrows in ***K–N*** denote diameter of the eyes. * denotes mild and ** severe hydrocephalus. The scale bar is 200 µm.

**Figure 3:**
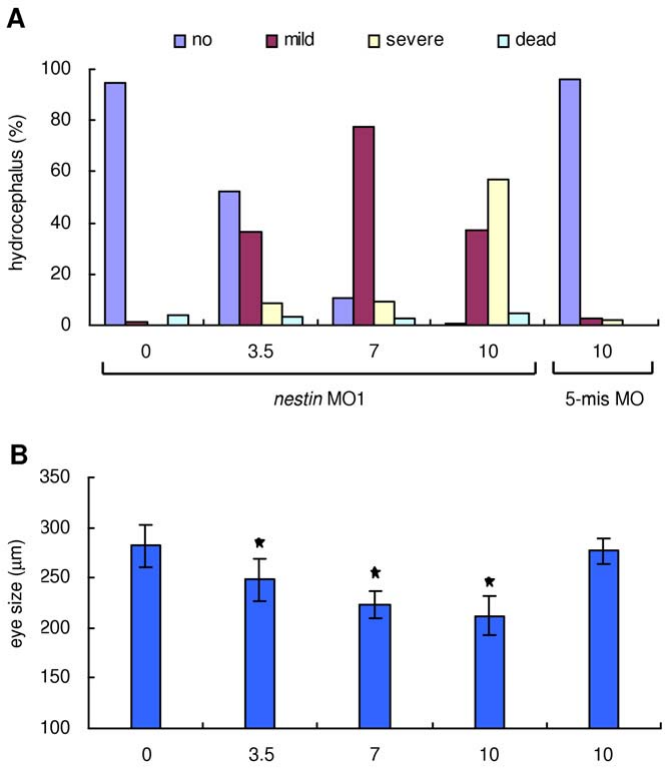
Dose-dependent induction of hydrocephalus and eye size changes by *nestin* MO1. ***A***. Hydrocephalus was visualized at 48 hpf after injecting increasing doses of *nestin* MO1 or 10 ng 5-mis *nestin* MO and classified as “mild” and “severe” as illustrated in Fig. 2M and 2N. Number of embryos (n) for each dose of *nestin* MO1 is: n = 125 for 0 group, n = 96 for 3.5 ng group, n = 125 for 7 ng group and n = 142 for 10 ng group. n = 118 for 5-mis MO group. ***B***. The diameter of eyes was measured at 48 hpf after *nestin* MO1 or 5-mis MO injection. Each bar represents mean ± SD. * denotes *p*<0.001.

The causal significance of *nestin* is established by specific trans-perturbation using morpholino-substituted antisense oligonucleotides. *Nestin* MO1 or MO2 (10 ng each) was microinjected into the yolk of one-cell stage zebrafish embryos and the phenotypes were examined at various time points. After knockdown of Nestin by either *nestin* MO, the embryos exhibited brain developmental defects at 24–28 hpf (Fig. 2A, C vs. 2B, D). By 51 hpf, the developmental defects had become pronounced (Fig. 2G, 2H and 2J), while neither control MO (Fig. 2E) nor 5-mis *nestin* MO (Fig. 2F) caused any morphological changes when compared to untreated wild-type embryos (data not shown). A magnified view of *nestin* MO-treated embryos at 51 hpf showed characteristic morphological abnormalities including small head, reduced brain size and hydrocephalus as well as small and poorly organized eyes (Fig. 2M and 2N). The hydrocephalus varied in size from mild (Fig. 2M) to severe (Fig. 2N). A dorsal view of *nestin* morphants revealed smaller tectum (Fig. 2J) than control (Fig. 2I). The morphological changes induced by *nestin*-MO persisted at 72 hpf (data not shown). Using hydrocephalus as an index of brain abnormalities, we found the effect of *nestin* MO on brain development was dose-dependent (Fig. 3A). Reduction of eye size was also dose-dependent (Fig. 3B). As treatment with 10 ng *nestin* MO1 induced hydrocephalus and eye changes in a majority of embryos (Fig. 3), we performed subsequent experiments by microinjection of 10 ng MO1.

**Figure 4:**
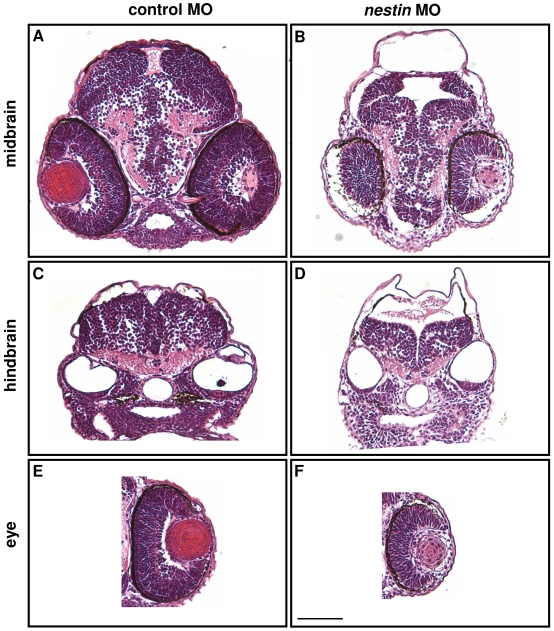
Examination of brain tissues and eyes by H&E staining. Embryos at 48 hpf after control or *nestin* MO injection were sectioned at midbrain (***A*** and ***B***) and hindbrain (***C*** and ***D***) levels. Tissues were stained with H&E. Eyes at the midbrain level were visualized and shown in ***E*** and ***F***. The scale bar is 50 µm.

Morphological changes of the brain and eyes in fish treated with 10 ng *nestin* MO were further evaluated by staining of the histological sections. H&E stained sections prepared from *nestin* MO-treated embryos show dilated ventricle, reduced tissue mass and defective organization in the midbrain (Fig. 4B) and hindbrain (Fig. 4D) and poorly organized retina and underdeveloped lens in both eyes (Fig. 4F). These results suggest that nestin is required for normal brain and eye development in zebrafish.

### Reduction of NPCs by Nestin MO Treatment

**Figure 5:**
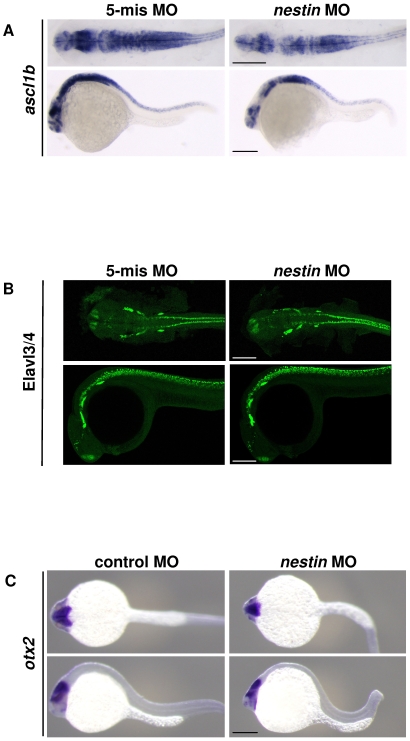
Effects of *nestin* MO injection on *ascl1b* (marker of NPC), Elavl3/4 (marker of mature neurons) and *otx2* (marker of midbrain tissue). ***A***. *Ascl1b* expression was analyzed by WMISH at 25 hpf. Upper panel shows dorsal view with deletion of yolk. Lower panel shows lateral view, head to the left. ***B***. Elavl3/4 was analyzed by confocal microscopy at 25 hpf. ***C***. *Otx2* was analyzed by WMISH at 25 hpf. Scale bar: 200 µm.

To identify neural cells affected by *nestin* MO, we performed (1) WMISH to detect *ascl1b*, a marker of NPCs; and *otx2*, a marker of midbrain neurons and (2) confocal microscopy to detect Elavl3/4, a marker of mature neurons. *Ascl1b* transcript was detected in the brain of zebrafish embryos at 25 hpf (Fig. 5A), and the transcript level was reduced in *nestin* morphant (Fig. 5A). Elavl3/4 was also detected in the brain and its expression pattern was not significantly altered by *nestin* MO treatment (Fig. 5B). *Otx2* was detected only in the midbrain and its expression was not different between *nestin* morphant and control (Fig. 5C). These results suggest that the effect of nestin knockdown was limited to NPCs.

**Figure 6:**
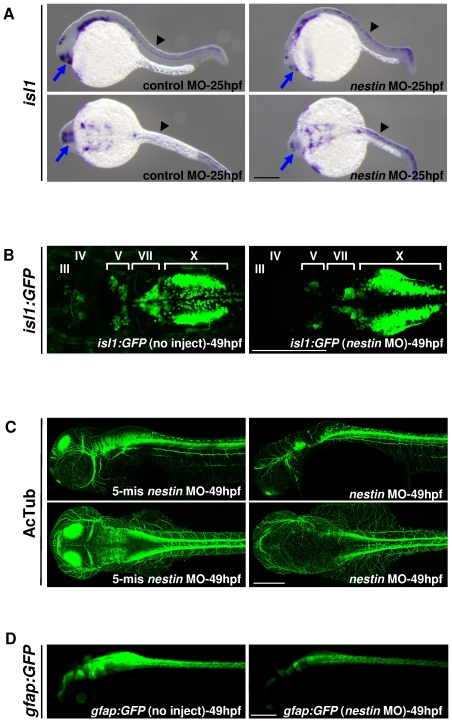
Effects of *nestin* MO injection on *isl1*, acetylated tubulin (AcTub) and *gfap:GFP* expression. ***A***. *Isl1* expression was analyzed by WMISH at 25 hpf. Arrow indicates for brain and arrow head, spinal cord. ***B***. Transgenic *Tg(isl1:GFP)* embryos were injected with *nestin* MO. GFP expression was examined under confocal microscopy at 49 hfp. Uninjected WT (no inject) was used as a control. ***C***. AcTub expression (axon marker) was examined by confocal microscopy at 49 hpf. The upper panels show lateral view and the lower panels, dorsal view. ***D***. Heterozygous *Tg(gfap:GFP)* embryos were injected with *nestin* MO and GFP was visualized under fluorescent microscopy at 49 hpf. Scale bars: 200 µm.

We next analyzed the expression pattern of motor neuron marker, *islet1*, using *in situ* hybridization and a transgenic fish line *Tg(isl1:GFP)*; glial lineage marker, glial fibrillary acidic protein (gfap) [15], using heterozygous *Tg(gfap:GFP)*, and axon marker, acetylated tubulin (AcTub), using immunofluorescence microscopy. Compared to control, *isl1* transcript in *nestin* morphant was reduced primarily in the forebrain (Fig. 6A) and increased slightly in the spinal cord (Fig. 6A). The results were highly reproducible in multiple experiments (Supplementary Fig. S1A and S1B). Analysis of cranial nerve *islet1* expression in *isl1-GFP* transgenic fish reveals that motor nerve III and IV were poorly detectable, and V and VII were reduced in size and disorganized in nestin morphants (Fig. 6B). There was a striking abnormality in nerve X morphology and positioning. Immunofluorescent microscopic analysis of AcTub expression reveals abnormal axon distribution (Fig. 6C). To evaluate the effect of *nestin* MO on *gfap* expression, we compared GFP intensity in heterozygous *Tg(gfap:GFP)* treated with *nestin* MO vs. control. There was a consistent reduction of GFP expression in *nestin* MO-treated embryos (Fig. 6D). These findings indicate the importance of nestin in motor neuron, axon and glial cell development.

### Increased Apoptosis in Nestin MO-Treated Embryos

**Figure 7:**
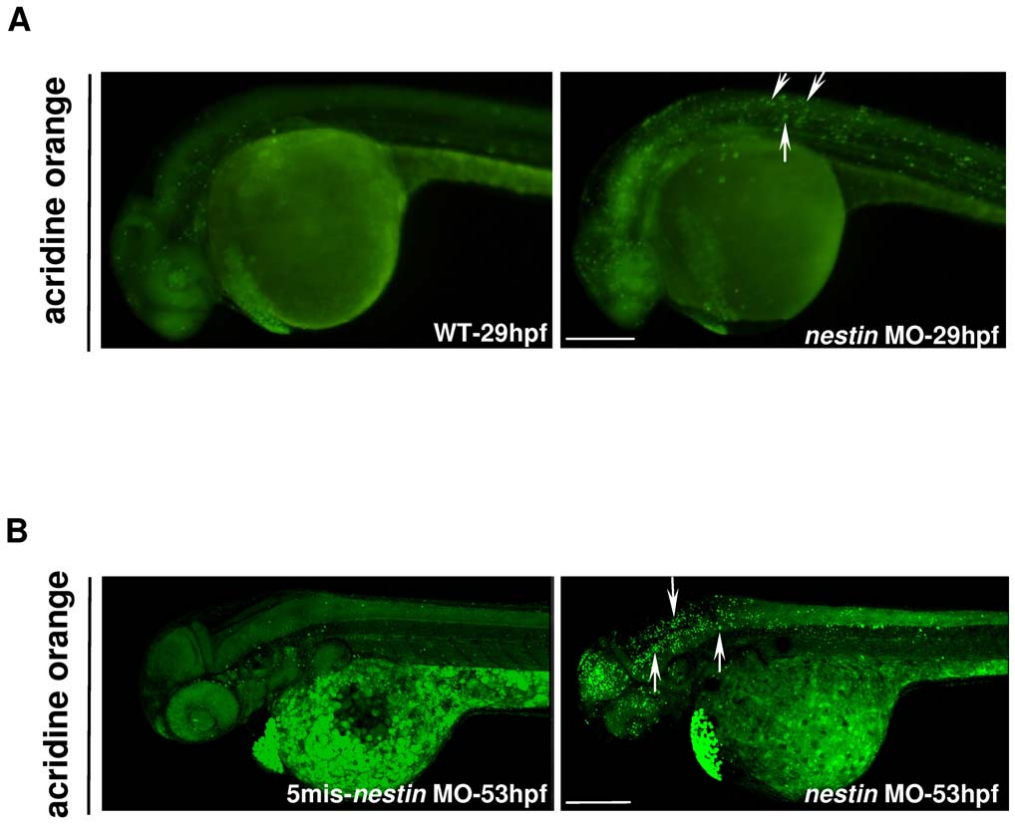
Analysis of apoptosis in live embryos with acridine orange staining. ***A***. Examination by stereomicroscopy at 29 hpf. ***B***. Examination by confocal microscopy at 53 hpf after injection of 10 ng of 5-mis or 10 ng of *nestin* MO. Arrows show positive staining.

**Figure 8:**
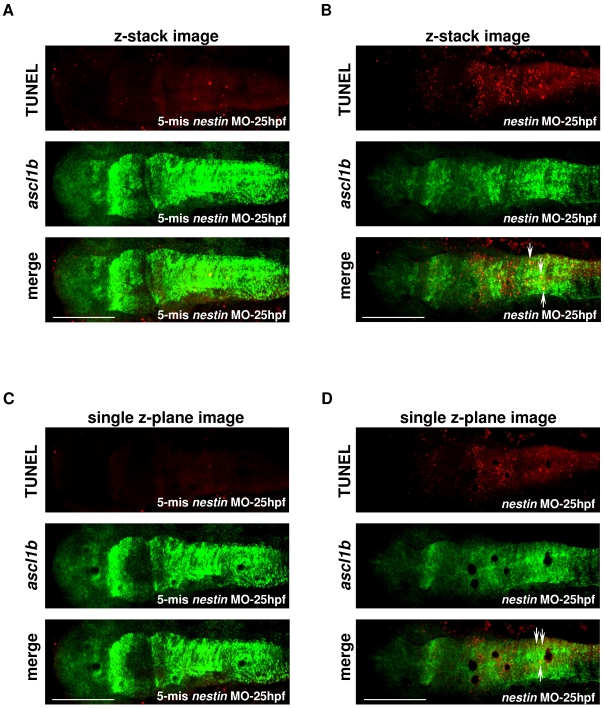
Colocalization of TUNEL signals with *ascl1b* expression. ***A*** and ***B***, z-stack images and ***C*** and ***D***, single z-plane images. Embryos were injected with (***A***) and (***C***) 5-mis *nestin* MO or (***B***) and (***D***) *nestin* MO and images were analyzed by confocal microscopy at 25 hpf. Top panels show TUNEL and middle panels, *ascl1b* stained by fluorescence *in situ* hybridization. The lower panels show merged images. Yellow dots as denoted by white arrows indicate colocalization of *ascl1b* with TUNEL. Bar scale: 200 µm.

The developmental defects in brain and eyes could be due to several possible mechanisms such as increased cell death and reduced cell proliferation. To assess cell death as a possible mechanism, we analyzed apoptotic cells in embryos by acridine orange staining and TUNEL assay. Acridine orange staining of living embryos revealed increased staining of brain cells especially the hindbrain region of *nestin* MO treated embryos at 29 hpf (Fig. 7A). The staining became more intense and extended to the midbrain and eyes at 53 hpf (Fig. 7B). Acridine orange staining was minimal in embryos treated with 5-mis *nestin* MO control (Fig. 7A and 7B). TUNEL-positive cells were examined by confocal microscopy. Analysis of z-stack images (Fig. 8A and 8B) revealed that TUNEL-positive cells were increased in the hindbrain of embryos treated with *nestin* MO for 25 hours (Fig. 8B, top panel) as compared to 5-mis *nestin* MO control (Fig. 8A, top panel). TUNEL-positive cells detected outside the brain are considered to be yolk debris. TUNEL-positive cells were noted to extend to the midbrain of embryos treated with *nestin* MO for 49 hours (Supplementary Fig. S2). To determine whether the apoptotic signals are localized to NPCs, we examined by fluorescence *in situ* hybridization *ascl1b* expression and merged the image with TUNEL. Analysis of the merged images shows increased numbers of cells in the hind brain exhibiting colocalization of TUNEL with *ascl1b* as indicated by increased yellow dots in Fig. 8B vs. Fig. 8A. To ascertain colocalization of TUNEL with *ascl1b*, we analyzed high magnification confocal images of a single z-plane (Fig. 8C and 8D). The results confirmed increased numbers of apoptotic *ascl1b*-positive cells in the hindbrain of embryos treated with *nestin* MO for 25 h (Fig. 8D vs. 8C). These results suggest that *nestin* MO increased apoptosis of *ascl1b* positive cells.

**Figure 9:**
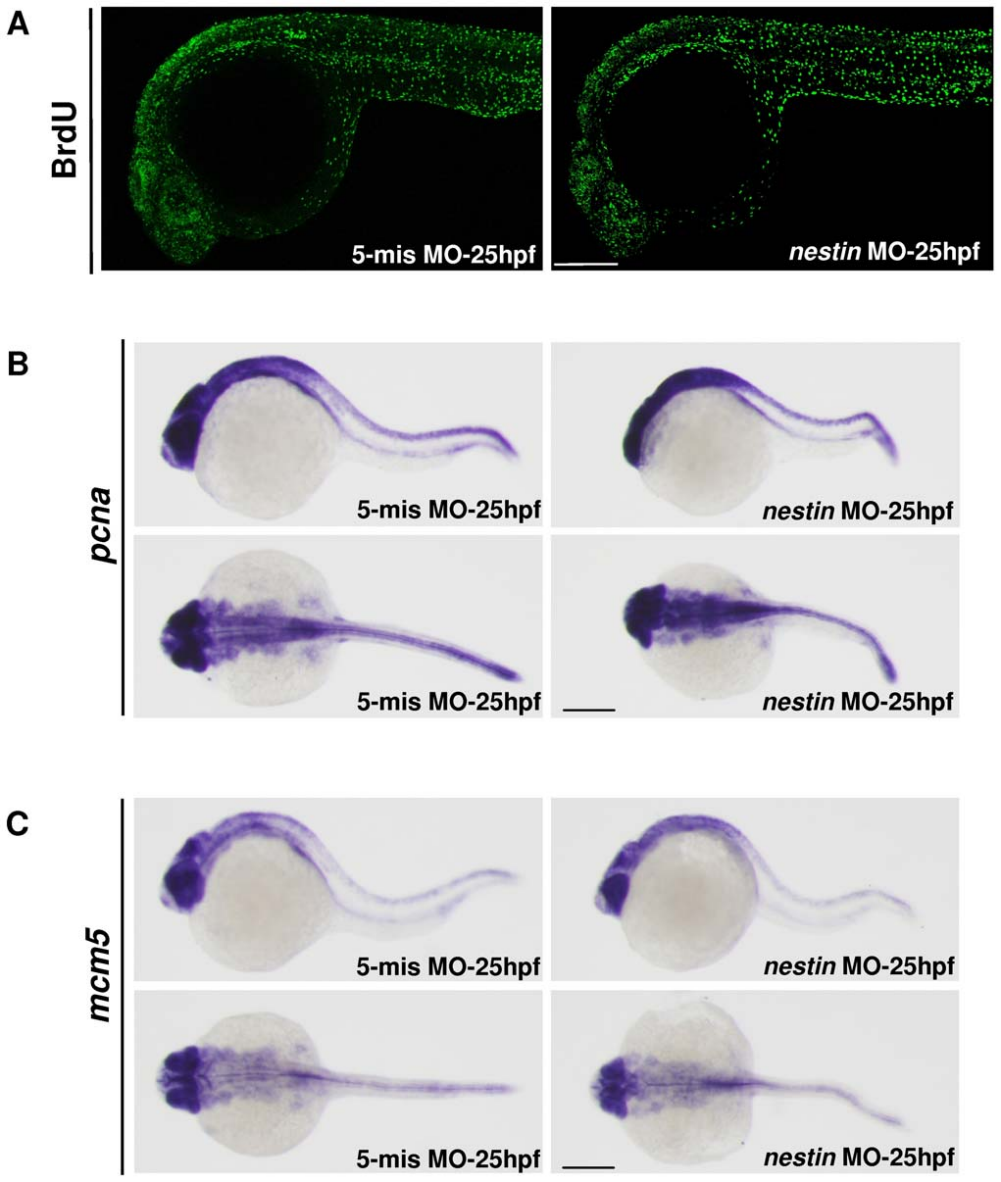
Influence of *nestin* MO on neural proliferation. ***A***. Analysis of BrdU incorporation in *nestin* MO vs. 5-mis MO-treated embryos at 25 hpf by immunofluorescence. ***B*** and ***C***. Analysis of (***B***) *pcna* and (***C***) *mcm5* transcripts by WMISH.

To determine whether knockdown of nestin reduces cell proliferation in brain and eyes, we analyzed BrdU uptake at 25 hpf. BrdU uptake was not significantly different between *nestin* MO and its 5-mis *nestin* MO control (Fig. 9A). Expression of proliferative markers such as *pcna* and *mcm5* was also not different between *nestin* MO and control (Fig. 9B and 9C).

## Discussion

*Nestin* is a hallmark of NPCs. Its expression in zebrafish embryos was first detected about 13 hpf and spread all over the brain and the spinal cord by 24 hpf. *Nestin* expression during this period of zebrafish embryo development may be critical for brain development. The findings reported here indicate that nestin is indeed essential for zebrafish brain and eye development. Knockdown of *nestin* in zebrafish embryo by morpholino injection causes distinct morphological changes characterized by small head, small eyes and overt hydrocephalus. These changes become more obvious with time. They are relatively mild at 24 hpf but become prominent at 48 hpf and thereafter. Histological examination of the brain and eyes of developing embryos confirms shrinkage of the hindbrain and the midbrain and the consequent enlargement of ventricles, as well as defective retina structure and underdeveloped lens. Severe suppression of Nestin protein expression at 24 hpf by *nestin* MO results in fewer NPCs in the brain as analyzed by *ascl1b*, a marker of NPCs in mammals and zebrafish [16], [17], [18]. However, *nestin* suppression does not reduce the density of mature neuron markers including Elavl3/4 and *otx2*. These results suggest that Nestin is required for the normal NPC function during embryo development.

Our results show developmental defects of motor neurons, axons and glial cells in *nestin* MO-teated embryos, indicating an important role of nestin in motor nerve and glial development. The underlying mechanism is not entirely clear. It was reported that *nestin* is expressed in a population of human parenchymal brain cells, co-expressing glial (*gfap*) and neuronal (*betaIII-tubulin*) markers. They differentiate into cells of the neuronal or the glial lineages through asymmetric regulation of gene expression. In neuronal cell differentiation, *nestin* and *gfap* are down-regulated while in glial cell differentiation, *betaIII-tubulin* and *nestin* are down-regulated [15]. Taken together, the results suggest that NPC *nestin* plays a pivotal role in determining the neuronal and glial cell specification.

Our data provide evidence that Nestin controls the survival of neuronal progenitor cells (NPC). We showed that loss of Nestin is associated with increased apoptosis of hindbrain and then midbrain cells. Colocalization experiments revealed that TUNEL-positive cells coincide with *ascl1b* expression. These results suggest that Nestin suppression enhances NPC apoptosis.

The use of MO in studying development may be limited by undesirable off-target effects that produce CNS developmental defects and apoptosis [19]. To minimize the chance of off-target effects, we have performed experiments such as dose-response, time course and use of multiple controls including a 5-nucleotide mutated *nestin* MO. The 5-mis MO control and a standard MO control up to 10 ng did not cause morphological abnormality and apoptotic changes. The results indicate that the defects in brain and eyes are unlikely due to off-target effects of MO as there is consistent dose-response and time-dependent effects.

Nestin is a selective NPC intermediate filament involved in cytoskeletal functions such as maintaining cell shape and regulating cell motility. Recent studies suggest that it is involved in other cellular functions [6]. Its role in apoptosis is less well characterized. However, several studies have indicated that the actin cytoskeleton regulates apoptosis and its defects are associated with neurodegenerative diseases [20]. Furthermore, filamentous aggregates of neuronal intermediate filament proteins are considered to be neurophathological signatures of neurodegenerative diseases [21]. It has been reported that deletion of the hepatocyte intermediate filament proteins, keratin 8/keratin 18 heterodimers, increases apoptosis [22]. Our results suggest that Nestin plays an important role in NPC survival in zebrafish embryo development. The mechanism by which nestin protects cell survival is unclear. Studies from murine cells have suggested that nestin may act as a scaffold to control cell functions including apoptosis [23]. It has been reported that in a murine NPC line, Nestin interacts with and sequesters cyclin-dependent kinase 5 (Cdk5), which is constitutively expressed in NPCs. Reduced Nestin protein levels lead to enhanced Cdk5 activity, promoting NPC apoptosis [23]. Further studies are needed to clarify whether control of NPC survival by Nestin in zebrafish is also mediated by this mechanism.

Our results show that the cranial motor nerve development is disrupted by suppression of *nestin* in *Tg(isl1:GFP)* fish which express GFP under the control of a motor neuron-specific *islet1* enhancer [12]. This model is well suited to detect changes in cranial motor nerves III, V, VII and X [24]. *Nestin* MO injection is accompanied by disappearance of III and IV, reduction of V and VII and aberrant positioning of X. These results indicate that the cranial motor neuron development is very vulnerable to *nestin* defects. Cranial axons are reduced or even abolished in *nestin* MO-treated embryos, most prominently in the tectum.

*Nestin* MO injection also has a profound effect on eye development. Both eyes are small and the retina and lens are underdeveloped in *nestin* knockdown fish. These developmental defects are correlated with increased retinal apoptosis. It has been shown that Nestin is expressed in murine retinal progenitor cells [25]. It is also reported in zebrafish that Nestin is expressed in the retinal ganglion cell layer and ciliary marginal zone, which constitutes the retinal proliferation zone [6]. Our whole mount *in situ* hybridization results are consistent with expression of *nestin* in embryonic eyes, presumably in the retinal progenitor cells. It is reasonable to assume that, like its role in neuronal development, Nestin is essential for the retinal development primarily by controlling the retinal progenitor cell survival. Lens development may require Nestin for similar reasons. Nestin was reported to be expressed in the epithelium of lens vesicle [26]. *Nestin* MO may induce apoptosis of the epithelial progenitor cells, resulting in defective lens development and organization.

In summary, nestin is widely expressed in developing zebrafish brain and eyes, especially in the neural, retinal and lens epithelial progenitor cells. It plays a crucial role in protecting progenitor cells from apoptosis thereby facilitating progenitor cell development into neurons, glial cells, motor nerves, retina and lens. Its deficiency leads to enhanced progenitor cell apoptosis and defective brain, eye and cranial nerve development.

## Supporting Information

Figure S1 Comparison of *isl1* expression in embryos treated with control MO vs. *nestin* MO. *Isl1* expression was analyzed by WMISH. Representative figures of four embryos are shown to illustrate reproducibility of the abnormalities described in Figure 6A. A. Lateral view. B. Dorsal view. Bar scale: 200 µm.
(3.36 MB EPS)

Figure S2 Analysis of apoptosis by TUNEL at 49 hpf. WT denotes uninjected wild-type embryo. Bar scale: 200 µm.
(3.80 MB EPS)
